# Antiviral Properties of *R. tanguticum* Nanoparticles on Herpes Simplex Virus Type I *In Vitro* and *In Vivo*

**DOI:** 10.3389/fphar.2019.00959

**Published:** 2019-09-04

**Authors:** Meng-xin Shen, Nian Ma, Min-ke Li, Yuan-yuan Liu, Tian Chen, Fei Wei, Dong-ying Liu, Wei Hou, Hai-rong Xiong, Zhan-qiu Yang

**Affiliations:** State Key Laboratory of Virology, Institute of Medical Virology, National Laboratory of Antiviral and Tumour of Traditional Chinese Medicine, Hubei Province Key Laboratory of Allergy and Immunology, School of Basic Medical Sciences, Wuhan University, Wuhan, China

**Keywords:** *R. tanguticum* nanoparticles, HSV-1, antiviral, mouse model, viral protein

## Abstract

Herpes simplex virus type 1 (HSV-1), an enveloped DNA virus, plays a key role in varieties of diseases including recurrent cold sores, keratoconjunctivitis, genital herpes and encephalitis in humans. Great efforts have been made in developing more effective and less side-effects anti-herpes simplex virus agents, including traditional Chinese herbal medicines. In the present study, we evaluated the antiviral efficacy of *Rheum tanguticum* nanoparticles against HSV-1 *in vitro* and *in vivo*. *R. tanguticum* nanoparticles could inactivate the HSV-1 virions and block the viral attachment and entry into cells. Time-of-addition assay indicated that *R. tanguticum* nanoparticles could interfere with the entire phase of viral replication. Besides, *R. tanguticum* nanoparticles showed the ability to inhibit the mRNA expression of HSV-1 immediate early gene *ICP4* and early gene *ICP8* as well as the expression of viral protein *ICP4* and *ICP8*. Moreover, *R. tanguticum* nanoparticles have been proved to protect mice against HSV-1 induced lethality by decreasing the viral load and alleviated pathological changes in brain tissues. In conclusion, we demonstrated that *R. tanguticum* nanoparticles could inhibit HSV-1 infection through multiple mechanisms. These results suggest that *R. tanguticum* nanoparticles may have novel roles in the treatment of HSV-1 infection.

## Introduction

HSV-1 is an enveloped, linear double-stranded DNA virus which is highly prevalent in most part of the world. Approximately 50–90% of the world’s population is seropositive for this virus (Smith and Robinson, 2002; Fatahzadeh and Schwartz, 2007). Diseases caused by herpes simplex virus include cold sores, keratoconjunctivitis, genital herpes and encephalitis (Fatahzadeh and Schwartz, 2007; Burcea et al., 2015; Sauerbrei, 2016). Treatments currently directed against HSV infections are nucleoside analogs such as acyclovir, valacyclovir, penciclovir, and famciclovir that target viral DNA polymerase (Vadlapudi et al., 2013). However, extensive use of these drugs has led to clinical problems with the emergence of drug-resistant virus strains, particularly in immunocompromised patients (Jiang et al., 2016). The discovery of new drugs to treat HSV infection has become an important goal of drug development.

Rhubarb is the rhizomes of plants that belong to the genus Rheum in the family Polygonaceae. Chinese Rhubarb includes *Rheum tanguticum* (*R. palmatum var. tanguticum*), *Rheum palmatum, Rheum officinale*, etc. The rhubarb contains several main chemical compositions such as anthraquinones, anthrones, stilbenes, tannins, polysaccharides, etc. (Cao et al., 2017). These compositions show a wide range of pharmacological activities, including antioxidant, anti-tumor, anti-microbial and anti-inflammatory activities (Lai et al., 2015; Cao et al., 2017). Moreover, anthraquinones derivatives like aloe-emodin, rhein, emodin, and chrysophanol, reportedly demonstrated antiviral effects (Semple et al., 2001; Lin et al., 2008; Schwarz et al., 2011; Wang et al., 2018). However, as with most Chinese herbal medicines, the application of rhubarb is limited due to its poor bioavailability and hydrophobicity. Therefore, it is necessary to find new ways for the usage of rhubarb in order to make better use of it. The field of nanotechnology is an advanced approach in modern materials science. Nanoparticles might resolve the biopharmaceutical problems related to improving the uptake of poorly soluble drugs, reducing toxicity and increasing the drug bioavailability (Onoue et al., 2014; Marella and Tollamadugu, 2018). Moreover, it is well documented that the unique properties of nanoparticles, such as small particle size, large surface area to volume ratios, and tunable surface charge, make nanoparticles attractive tools for viral treatment (Singh et al., 2017). In recent years, several nanoparticles have been reported for the treatment of viral infections, among them silver nanoparticles have proven to be active against several types of viruses (Galdiero et al., 2011; Singh et al., 2017).

Given the importance of antiviral effect of rhubarb and advantages of nanoparticles, we designed some assays to investigate the activity of *R. tanguticum* nanoparticles against herpes simplex virus type I. We first conducted plaque reduction assays using HEp-2 cells to test the capacity of these nanoparticles to inactivate the HSV-1 virions and block the viral attachment and entry into cells, following the evaluation of inhibitory effect on viral replication using real-time quantitative PCR, Western blot, and immunofluorescence methods. Furthermore, the *in vivo* efficacies of these nanoparticles were investigated with a mouse model of HSV-1 encephalitis. The positive results offer a novel promising way for the usage of Chinese herbal medicine to control the HSV-1 infection.

## Materials and Methods

### Preparation of *R. tanguticum* Nanoparticles

*R. tanguticum* were collected from some high mountainous areas in Qinghai provinces, China. The plant was authenticated by Prof. Keli Chen, Department of Identification and Assessment of TCM, Hubei University of Chinese Medicine (Hubei, China). A voucher specimen (RQ/20140315) was deposited in School of Basic Medical Sciences, Wuhan University. *R. tanguticum* nanoparticles were produced from Shenzhen Lvwa Biological Technology Co., Ltd. The dried *R. tanguticum* roots were grounded to produce coarse powders using medicinal herb grinder (CW700, Shandong, China). Then the coarse powders were passed through a 100 mesh sieve, mixed with a quantity of pure water and subjected to a gel grinding (JMS-240, Hebei, China) with wet grinding to the required particle size less than 50 µm. Then these mixed particles were smashed to cell breaking as nanoparticles using the high pressure homogenizer (APV-2000, Germany). Finally, the *R. tanguticum* nanoparticles were obtained by drying process before usage.

### Analysis of the Active Ingredients of *R. tanguticum* Nanoparticles

The content of main components of *R. tanguticum* nanoparticles was performed by high-performance liquid chromatography (HPLC). Chromatography analysis was performed on a Waters e2695 Alliance HPLC system equipped with an Agilent Zorbax SB-C18 column (150 mm × 4.6 mm, 3.5 µm). The mobile phase composed of A) methanol and B) 0.1% (v/v) phosphoric acid water solution was delivered at a rate of 1 ml/min. The reference marker compounds of aloe-emodin, rhein, emodin, chrysophanol, and physcion for high-performance liquid chromatography (HPLC) assay were purchased from the Shanghai Standard Technology Co., Ltd. (Shanghai, China) and the purity of all these compounds were higher than 98.0%.

### Morphology Observation by Transmission Electron Microscopy

*R. tanguticum* nanoparticles were characterized by a transmission electron microscope (TEM, HT7700, Hitachi). A drop of *R. tanguticum* nanoparticles suspension was placed on a carbon coated-copper grid and evaporated to form a monolayer at room temperature and then mounted on a specimen stub in the transmission electron microscope. The elemental analysis was carried out with an accelerating voltage of 80 kV.

### Evaluation of Particle Size and Zeta Potential

The particle size distribution of *R. tanguticum* nanoparticles was measured via dynamic light scattering (DLS) technique using a Nano ZS90 Zetasizer (Malvern Instruments, Malvern, UK) at 633 nm. The zeta potential (ZP) of *R. tanguticum* nanoparticles was measured with the same instrument. The samples were suspended in distilled water and were analyzed in triplicate at a temperature of 25°C.

### Cells, Virus and Plant Material

HEp-2 (human laryngeal carcinoma) cells were grown in Dulbecco’s modified Eagle’s medium (DMEM) (Gibco, Grand Island, NY) supplemented with 10% fetal bovine serum (Gibco), 100 U/ml penicillin (Gibco) and 100 µg/ml streptomycin (Gibco) at 37°C in 5% CO_2_. The virus strains used were HSV-1 F gifted by Professor Rapp. F from Hershey Medical Center, USA in 1990 and stored in our laboratory ever since. The virus was propagated in HEp-2 cells and stored at −80°C for further studies. *R. tanguticum* nanoparticles were dissolved in (DMSO) (Sigma-Aldrich, St Louis, MO) followed by dilution in Dulbecco’s modified Eagle’s medium (DMEM) (Gibco, Grand Island, NY) to prepare a stock concentration of 1 mg/ml solution.

### Cytotoxicity Assay

The 50% cytotoxic concentration (CC_50_) of *R. tanguticum* nanoparticles on HEp-2 cells was performed by MTT assay (Shi et al., 2007). HEp-2 cells in 96-well plates were incubated with various concentrations of *R. tanguticum* nanoparticles (0, 31.25, 62.50, 125, 250, 500, and 1,000 µg/ml) at 37°C in an atmosphere of 5% CO_2_. After incubation, an MTT assay (Sigma-Aldrich) was performed according to the manufacturer’s protocol. The optical density at 570 nm was then determined by a conventional microplate reader (Multiskan^TM^ GO; Thermo Fisher Scientific, Waltham, MA). The CC_50_ was calculated using a regression analysis.

### Plaque Reduction Assay

The antiviral activity was performed using the plaque reduction assay (PRA) as previously described (Prichard et al., 1990). Briefly, HEp-2 cell monolayers seeded on 24-well plates were infected with HSV-1 (100 PFU/well). After 2 h incubation, the cells were washed twice with pre-warmed PBS and overlaid with 1% methylcellulose containing different concentrations of *R. tanguticum* nanoparticles as indicated. The plaques developed after 72 h of incubation were fixed with 4% paraformaldehyde for 10 min and were stained with 1% crystal violet solution. The plaques were counted and the percentages of inhibition were calculated. The effective concentration of test compound that reduced plaques number by 50% (EC_50_) was calculated.

### Time of Addition Assay

Time of addition assay was performed as described previously (Bertol et al., 2011). Briefly, 350 µg/ml *R. tanguticum* nanoparticles were added to HEp-2 cell monolayers at indicated time periods after HSV-1 infection (MOI = 0.01). After 72 h incubation at 37°C, total HEp-2 cells were collected and frozen and thawed for three times. Virus titer was determined by plaque assay.

### Virus Inactivation Assay

Equal volumes of diluted virus (2.0 × 10^5^ PFU/ml) were mixed with each drug dilution and were incubated at 37°C for 1 h. The mixture was then 1,000 times diluted with fresh DMEM containing 2% fetal bovine serum (FBS) to yield a sub-therapeutic concentration of the test compound and to generate 100 PFU/ml HSV-1. The virus inocula were then added to HEp-2 cell monolayers. After adsorption for 2 h, the viral inoculum were discarded, the infected cells were washed once with PBS, and then overlaid with overlay media. Plates were incubated at 37°C for 72 h and were subjected to the plaque assay, as described previously (Lin et al., 2011).

### Attachment and Penetration Assays

The attachment and penetration assays were performed as described previously (Gong et al., 2002) with minor modifications. For the attachment assay, HEp-2 cell monolayers were pre-chilled at 4°C for 1 h. The medium was replaced with different concentrations of cold *R. tanguticum* nanoparticles and was incubated for 2 h at 4°C. Then, 100 PFU of virus was added to the cells. After infection for 2 h, the unbound virus was removed. The cells were washed twice with cold PBS and were covered with methylcellulose to allow plaque formation. For the penetration assay, HEp-2 cell monolayers were cooled to 4°C for 1 h and then incubated with 100 PFU of HSV-1 at 4°C for 2 h to allow viral adsorption. The unbound viruses were removed and HEp-2 cells were incubated with different concentrations of *R. tanguticum* nanoparticles at 37°C for 2 h to facilitate viral penetration. At the end of the incubation, the cells were washed with PBS, acidic glycine (pH 2.2) for 1 min, and DMEM (no serum), respectively. Then the cells were overlaid with methylcellulose for virus plaque formation.

### Real-Time Quantitative PCR (RT-qPCR)

HEp-2 cells were infected with HSV-1 at an MOI of 0.01 and were treated with *R. tanguticum* nanoparticles (350 µg/ml) at 6, 12, 18, and 24 h intervals, and total RNA was extracted with TRIzol reagent (Ambion^®^, Life Technologies Pty Ltd., Carlsbad, CA) according to the manufacturer’s instructions. The cDNA was synthesized in a reverse-transcriptase reaction by using Thermo cDNA Synthesis Kit (Thermo Scientific, Rockford, IL). The immediate early gene α4 (encoding ICP4), early gene *U* *_L_* *29* (encoding ICP8) and late gene *U* *_S_* *6* (encoding glycoprotein gD) were determined by using a Bio-Rad CFX96 instrument according to a two-step protocol (95°C for 2 min, 45 cycles of 5 s at 95°C, 10 s at 60°C, and 15 s at 72°C). The transcription levels of total RNA in each sample were standardized against the GAPDH gene. The sequences of Primers used were as follows: ICP4 (5’-CGACACGGATCCACGACCC-3’ and 5’-GATCCCCCTCCCGCGCTTCGTCCG-3’);ICP8 (5’-CGACAGTAACGCCAGAAG-3’ and 5’-GGAGACAAAGCCCAAGAC-3’); gD(5’-GCCCCGCTGGAACTACTATG-3’ and 5’-TTATCTTCACGAGCCGCAGG-3’);GAPDH(5’-GGTGGTCTCCTCTGACTTCAACA-3’ and 5’-GTTGCTGTAGCCAAATTCGTTGT-3’). Relative quantification was carried out based on the 2^−ΔΔCT^ threshold cycle method.

### Immunofluorescence Assay (IFA)

The HSV-1(MOI = 0.01) infected HEp-2 cells were in the presence or absence of *R. tanguticum* nanoparticles (350 µg/ml). At 6, 12, 18, and 24 h.p.i., the cells were fixed with pre-chilled 4% paraformaldehyde for 30 min and were permeabilized in PBS containing 0.25% Triton X-100 for 10 min at room temperature and then rinsed thrice in PBS, following by blocked in 1% BSA for 30 min. Cells were further washed with PBS, then incubated for 2 h at 37°C with anti-ICP4 antibody (Abcam, ab6514, 1:1000) and anti-ICP8 (Abcam, ab20194, 1:500) antibody, respectively. The cells were then rinsed three times for 5 min in PBS, followed by incubation with the 488-conjugated goat anti-mouse IgG (H+L) secondary antibody (diluted 1:200) for 60 min at 37°C and counterstained with DAPI. Images were observed using a fluorescence microscope (TE2000; Nikon, Tokyo, Japan) (Roberts and Baines, 2011).

### Western Blot Analysis

HEp-2 cells were infected with HSV-1 at an MOI of 0.01 and were incubated with or without *R. tanguticum* nanoparticles (350 µg/ml) at intervals of 6, 12, 18, and 24 h post-infection. Cells were harvested in RIPA Lysis Buffer (Biosharp, Hefei, China) and the soluble fraction was then clarified by centrifugation at 12,000 × g for 5 min at 4°C. Equal amounts of protein (40 µg/sample) were then isolated by 8% sodium dodecyl sulphate polyacrylamide gel electrophoresis (SDS-PAGE) and transferred to a pre-equilibrated PVDF membrane (Thermo Scientific). Membranes were blocked with 5% BSA for 2 h, rinsed and followed by incubation with anti-ICP4 antibody (Abcam, ab6514, 1:1000), anti-ICP8 antibody (Abcam, ab20194, 1:500) and anti-GAPDH antibody (Tianjin Sungene Biotech KM9002T, Beijing, China, 1:5,000) in 5% BSA at 4°C overnight, respectively. The membranes were washed and incubated with horseradish peroxidase-conjugated secondary antibodies for 2 h at room temperature and visualized by using an ECL Western Blot Detection Kit (Millipore Corp., Bedford, MA).

### Animal Experiment Design

Male Kunming mice (weight: 15–17 g) were purchased from the Animal Research Center of Wuhan University (Certificate No. SCXK 2015-0018, Hubei, China). The animal study protocols were approved by the Institutional Animal Care and the Ethics Committee of Wuhan University School of Medicine. Mice were anesthetized intraperitoneally by ketamine (100 mg/kg) and injected intracerebrally with 20 μl 10-fold serial dilutions of HSV-1(HSV-1 F strain). The 50% lethal dose (LD_50_) of virus was calculated by Reed-Muench method (Ling et al., 2012). Clinically, according to human dosage of rhubarb recommended in Chinese Pharmacopoeia (Commission, 2015), the equal doses of 3–15 g should be suggested to 0.46–2.28g/kg of mice. We set *R. tanguticum* nanoparticles groups with five doses of 2,500 mg/kg/day, 1,250 mg/kg/day, 625 mg/kg/day, 312.5 mg/kg/day, 156.3 mg/kg/day to determine the drug toxicity in mice. No death and obvious weight loss were seen when *R. tanguticum* nanoparticles were at dosages below 2,500 mg/kg/day. The daily animal doses of 312.5 and 625 mg/kg/day of *R. tanguticum* nanoparticles used in the study corresponded to doses of 2.06 and 4.12g of rhubarb translated from the clinical dosage for an adult human (60 kg) (Reagan-Shaw et al., 2008).

To investigate the protective activity of *R. tanguticum* nanoparticles against HSV-1 *in vivo*, the mice were randomly assigned to 5 groups and inoculated intracerebrally with 20 μl of DMEM (Gibco, Grand Island, NY) containing either no virus (vehicle only) or 5LD_50_ of HSV-1(HSV-1 F strain). After 5 h, the inoculated mice received the following treatment: *R. tanguticum* nanoparticles were dissolved in Saline (0.9%) before administration to mice with the indicated doses at 625 mg/kg/day or 312.5 mg/kg/day, respectively; the positive controls received 100 mg/kg/day acyclovir. Saline (0.9%) was used in normal and viral controls. The drugs were administered *via* oral gavage once daily or acyclovir twice daily at 12 h intervals for five consecutive days (Quenelle et al., 2010).

For survival curve experiments, 10 mice per group were observed for mortality and weighed daily for 14 days. The protection was estimated by body weight evaluation, the survival time and the reduction of mortality. For viral titer analysis, four mice per group were sacrificed on day 5 post infection. Brains were then harvested and subsequently homogenized to 10% (w/v) suspensions in test medium. The homogenates were frozen and thawed twice to release the virus and centrifuged and the supernatant was used to test the virus titers in the HEp-2 cells by plaque assay. For histological examination, on day 5 post infection, brains of mice were removed and fixed (n = 4/group) with 4% paraformaldehyde, and then embedded in paraffin, sectioned, and stained with haematoxylin and eosin (H&E). For gene expression and protein levels assays, brains of four mice per group were collected on day 5 post infection and total RNA was extracted from brain tissues with TRIzol reagent following the manufacturer’s instructions. The expression of two viral genes (*ICP4* and *ICP8*) was determined using RT-qPCR. Furthermore, the *ICP4* proteins of the treated and untreated mice with virus infection were analyzed using Western blot as previously described with minor modifications (Menasria et al., 2013).

### Statistical Analysis

Data were presented as the mean ± standard deviation (mean ± SD) and analyzed with the GraphPad Prism software v.5 (GraphPad Software Inc., La Jolla, CA). Statistic differences between groups were determined by one-way analysis of variance (one-way ANOVA) with Bonferroni’s multiple comparison tests. The probability of the mouse survival was estimated using Log-rank test. A value of *p* < 0.05 was considered significant.

## Results

### HPLC Profile of *R. tanguticum* Nanoparticles

**Figure 1:**
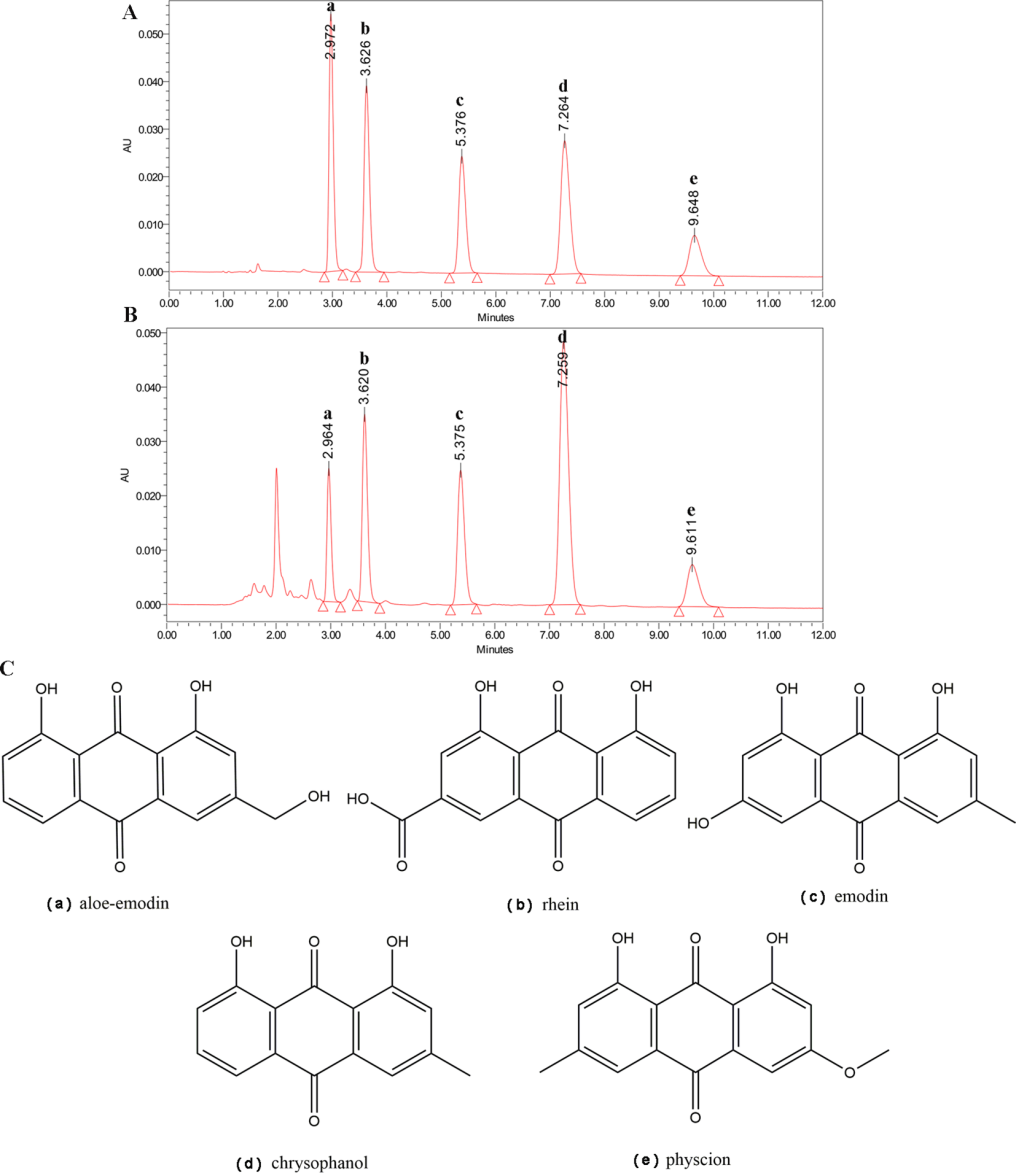
HPLC analysis of *R. tanguticum* nanoparticles. **(A)** HPLC of the standard reference compounds. **(B)** The chemical contents of *R. tanguticum* nanoparticles, peak a for aloe–emodin, peak b for rhein, peak c for emodin, peak d for chrysophanol, and peak e for physcion, respectively. **(C)** Structure of identified components of *R. tanguticum* nanoparticles.

To determine the main chemical profile of the *R. tanguticum* nanoparticles, the five bioactive constituents of *R. tanguticum* nanoparticles, the aloe-emodin, rhein, emodin, chrysophanol, and physcion were quantified using HPLC in comparison with standard reference compounds (**Figure 1**). The concentrations of aloe-emodin, rhein, emodin, chrysophanol, and physcion in *R. tanguticum* nanoparticles were 0.226, 0.443, 0.503, 0.853 and 0.252%, respectively. The result revealed that the content of the five anthraquinone compounds in *R. tanguticum* nanoparticles was in accord with the quality standard of Chinese Pharmacopoeia (Commission, 2015).

### Characterization of *R. tanguticum* Nanoparticles

**Figure 2:**
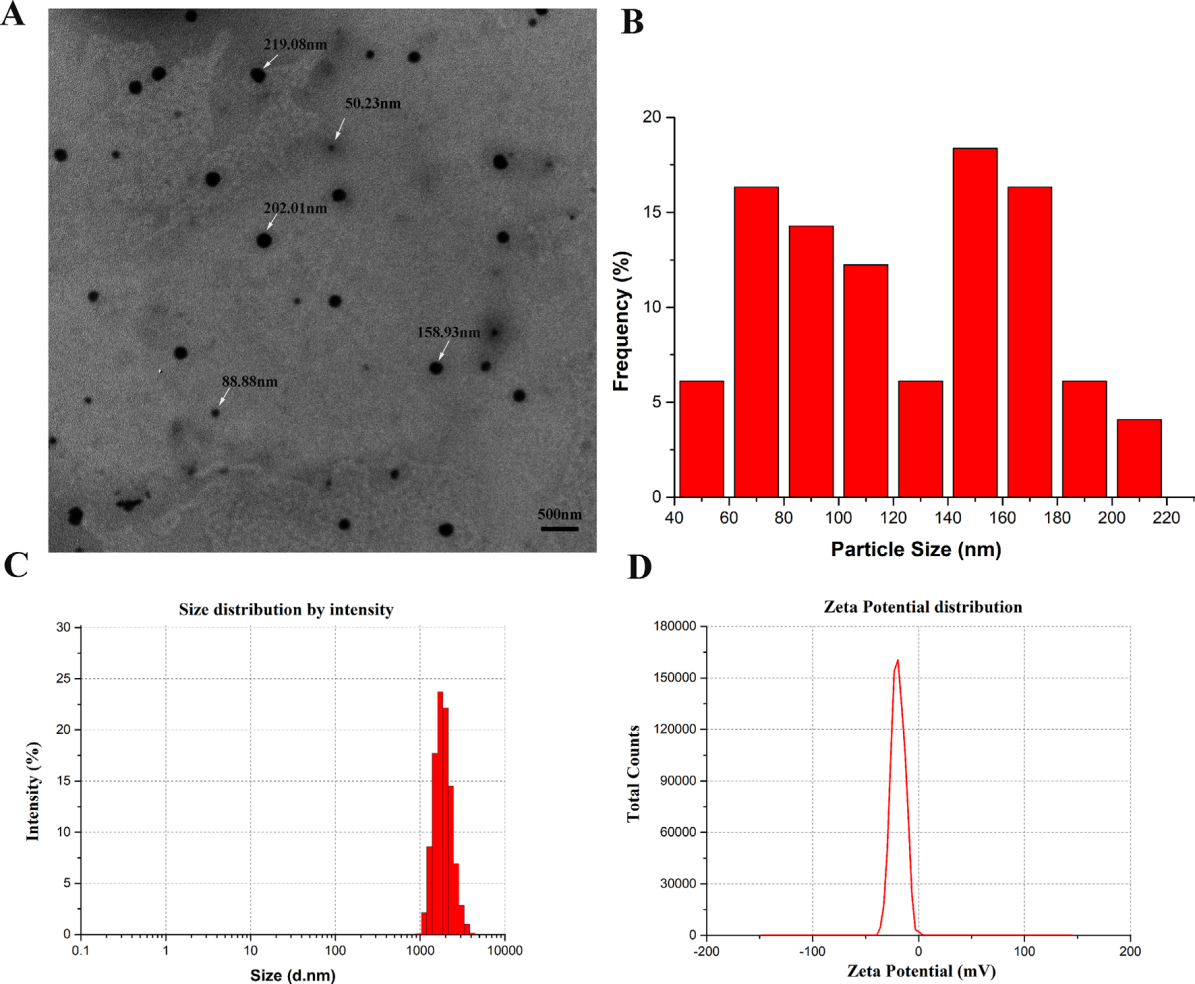
Characterization of *R. tanguticum* nanoparticles **(A)** Transmission electron microscopy (TEM) images of *R. tanguticum* nanoparticles. **(B)** TEM size distribution histogram. **(C)** Dynamic light scattering (DLS) size distribution histogram. **(D)** Zeta potential distribution of *R. tanguticum* nanoparticles.

The morphology and size of *R. tanguticum* nanoparticles were characterized by using TEM and DLS techniques. Transmission electron microscopy (TEM) images showed the *R. tanguticum* nanoparticles distributed in spherical shape, with sizes ranging from 50 to 219 nm (**Figure 2A**). The mean particle size of *R. tanguticum* nanoparticles calculated by TEM was around 123.2 nm (**Figure 2B**). The average hydrodynamic size of *R. tanguticum* nanoparticles determined by DLS was found to be 1794.7 ± 21.7 nm (**Figure 2C**). Polydispersity index (PDI) and zeta potential were 0.13 ± 0.05 and −19.93 ± 0.38 mV, respectively (**Figure 2D**). The small Polydispersity index (PDI) value, which is less than 0.3, indicates the *R. tanguticum* nanoparticles are uniform in particle size with a narrow particle size range (Yuan et al., 2008). The zeta potential values were a little low which may induce agglomeration in nano system (Gan et al., 2005). While the particle size measured by DLS showed a much larger diameter than the TEM observation, probably because of the *R. tanguticum* nanoparticles tend to aggregate in aqueous state.

### Inhibitory Effect of *R. tanguticum* Nanoparticles on HSV-1 Replication

**Figure 3:**
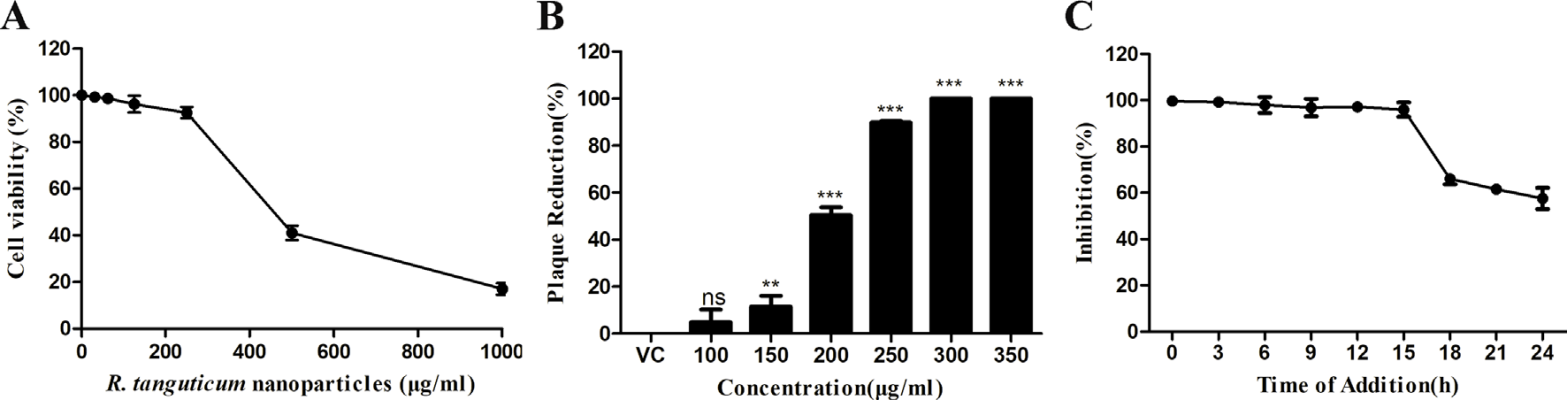
*R. tanguticum* nanoparticles inhibition of HSV-1 replication. **(A)** HEp-2 cells in 96-well plates were treated with serial dilution of *R. tanguticum* nanoparticles for 72 h and the viability were measured by the MTT assay. **(B)** Inhibitory effects of *R. tanguticum* nanoparticles on HSV-1 infection. HEp-2 cells infected with HSV-1 were treated with different concentrations of *R. tanguticum* nanoparticles and were subjected to the plaque reduction assay. **(C)** Time-of-addition assay. HEp-2 cells were infected with HSV-1 and then treated with *R. tanguticum* nanoparticles (350 µg/ml) at indicated intervals. The progeny virus yields were determined by plaque assay. Values are represented as the mean ± standard deviation of three individual experiments. “VC” is the abbreviation of the “virus control group”.*p < 0.05; **p < 0.01; ***p < 0.001; n.s., not significant.

**Figure 4:**
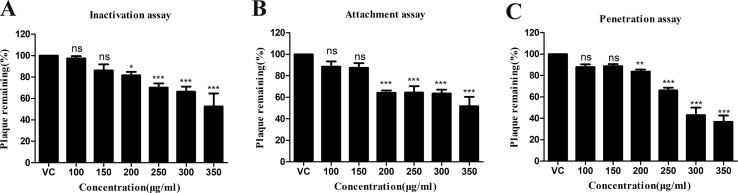
The anti-inactivation **(A)** anti-attachment **(B)** and anti-penetration **(C)** activities of *R. tanguticum* nanoparticles. Values are represented as the mean ± standard deviation of three individual experiments. “VC” is the abbreviation of the “virus control group”.*p < 0.05; **p < 0.01; ***p < 0.001; n.s., not significant.

In order to evaluate the antiviral efficacy of *R. tanguticum* nanoparticles on HSV-1 infectivity, we first determined their cytotoxicity by MTT assays. HEp-2 cells were treated with serial dilutions of *R. tanguticum* nanoparticles at 37°C for 72 h. As shown in **Figure 3A**, the 50% cytotoxic concentration (CC_50_) of *R. tanguticum* nanoparticles on HEp-2 cells was 415.3 µg/ml. Thus, a safe and low-toxicity concentration 350 µg/ml was selected to determine the anti-HSV-1 efficacy of *R. tanguticum* nanoparticles using a plaque reduction assay (PRA). The anti-HSV-1 efficacy of *R. tanguticum* nanoparticles was further conducted using a plaque reduction assay (PRA). The results showed that *R. tanguticum* nanoparticles could suppress HSV-1 viral plaque formation in a dose-dependent manner (**Figure 3B**) and with the increase of drug concentrations, a noted increased antiviral activity was observed. The EC_50_ of *R. tanguticum* nanoparticles determined by PRA was 194.1 µg/ml. To investigate the activity of *R. tanguticum* nanoparticles on viral replication cycle, time-of-addition assay was performed (**Figure 3C**). *R. tanguticum* nanoparticles (350 µg/ml) were added to HEp-2 cell monolayers at indicated times post-infection (h.p.i.) and the cells were harvested at 72 h.p.i. The inhibitory effect was significant when *R. tanguticum* nanoparticles were added at 0–15 h.p.i. Moreover, the results of the inactivation, attachment and penetration assays suggested that *R. tanguticum* nanoparticles also show effect on directly inactivate HSV-1 particles, prevent viral adsorption and block HSV-1 entry into cells. As shown in **Figure 4**, compared with those in the virus control, the 200–350 µg/ml concentrations of *R. tanguticum* nanoparticles inhibited both the inactivation, attachment and penetration processes of HSV-1 and the viral activities were dramatically reduced. These findings suggested that the antiviral activity of *R. tanguticum* nanoparticles was due to the interference with the whole phase of viral replication.

### *R. tanguticum* Nanoparticles Inhibit the Expression of HSV-1 Immediate Early, Early, and Late Genes

**Figure 5:**
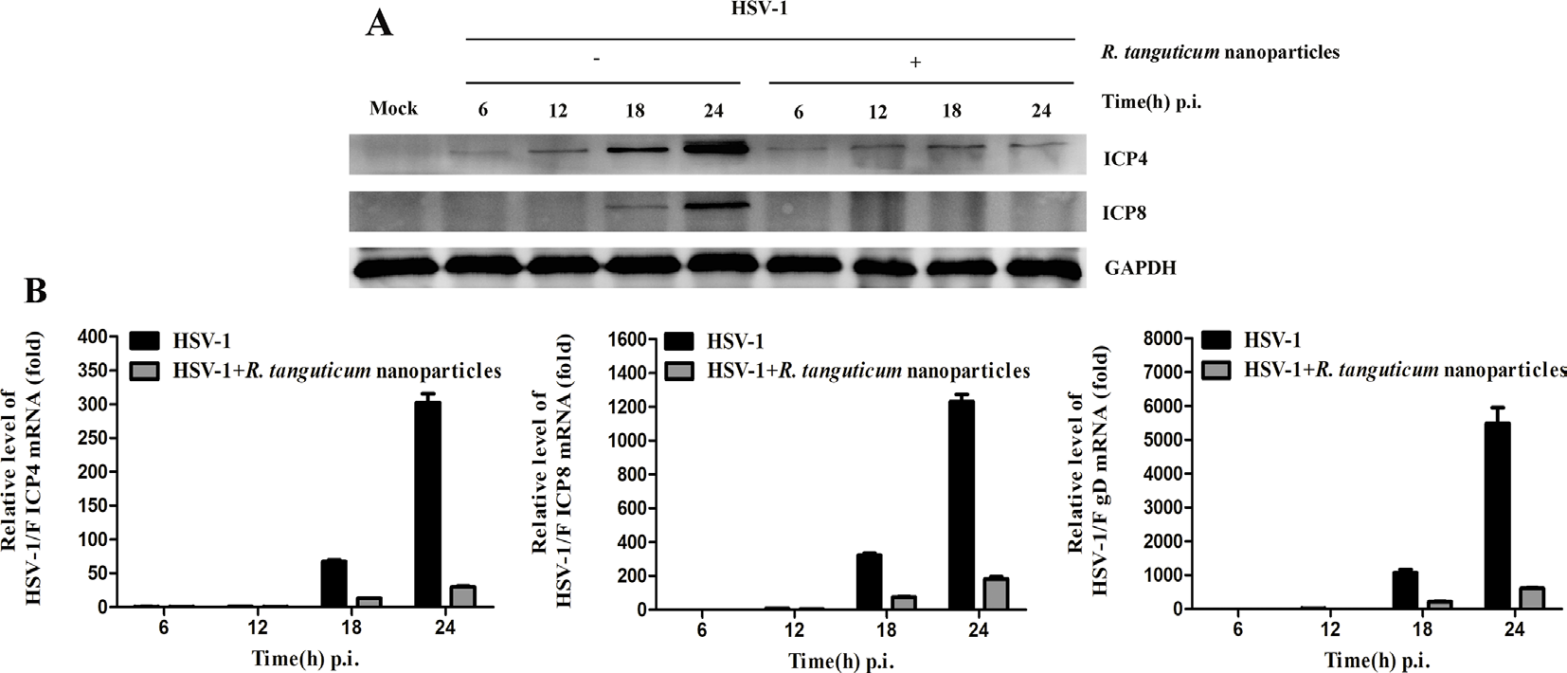
The effect of *R. tanguticum* nanoparticles on HSV-1 immediate-early and early genes expression. **(A)** Western blot analysis: HEp-2 cells were infected with HSV-1 at an MOI of 0.01 and then treated with or without 350 µg/ml *R. tanguticum* nanoparticles. The cells were harvested at each time point (6, 12, 18, and 24 h) for western blot analysis of *ICP4* and *ICP8*. **(B)** Real-time quantitative PCR analysis: HSV-1 (MOI = 0.01) infected cells were treated with 350 µg/ml *R. tanguticum* nanoparticles for 6, 12, 18, and 24 h.p.i. post-infection. Total RNA was extracted and subjected to cDNA synthesis. The real-time quantitative PCR was performed *ICP4*-, *ICP8*-, and *gD*-specific primers. Values are represented as the mean ± standard deviation of three individual experiments.

**Figure 6:**
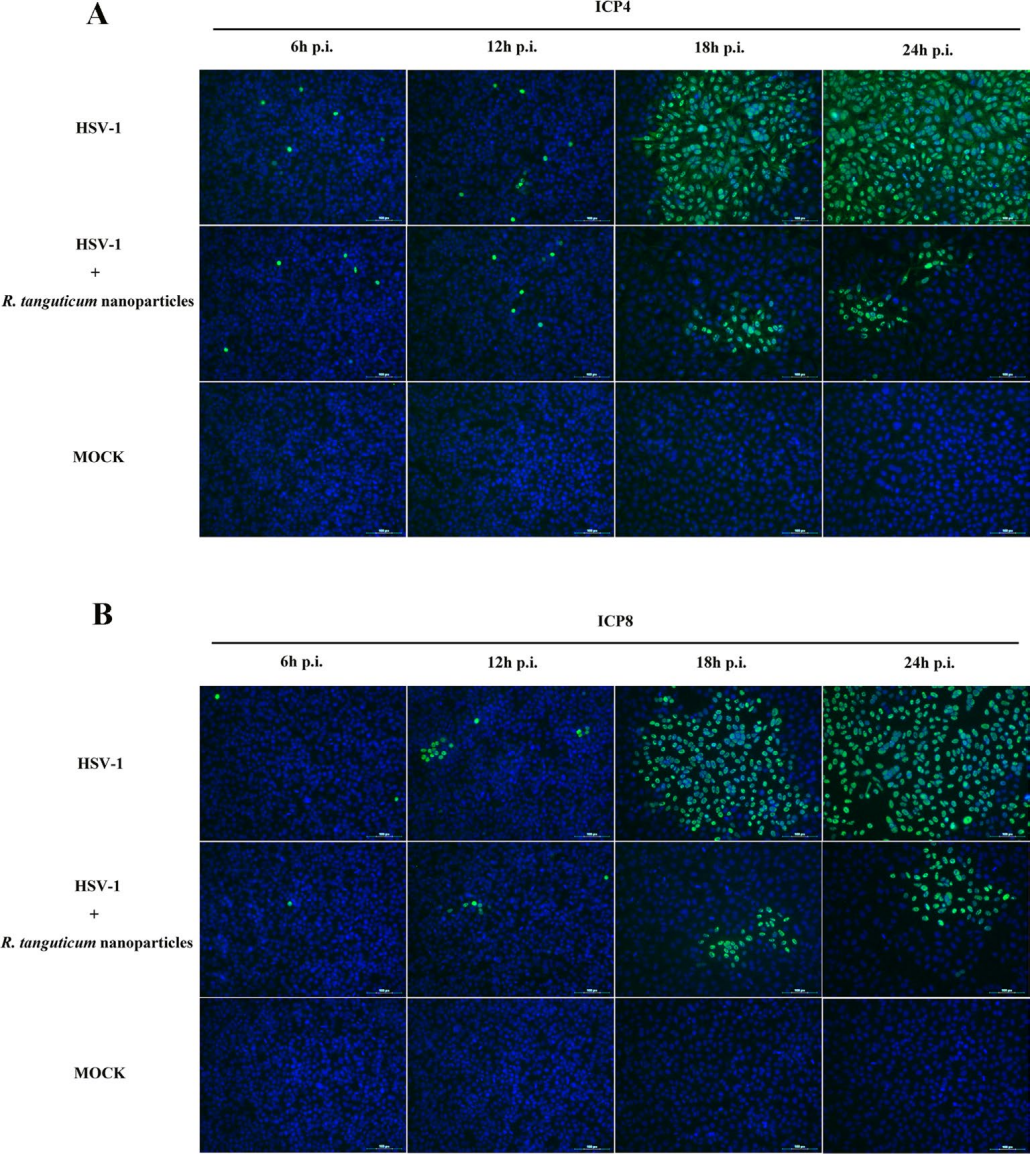
Immunofluorescence assay of HSV-1 infected cells treated with *R. tanguticum* nanoparticles. **(A–B)** HEp-2 cells were infected with HSV-1 at an MOI of 0.01 and incubated with 350 µg/ml *R. tanguticum* nanoparticles. At 6, 12, 18, and 24 h.p.i., cells were fixed with paraformaldehyde and blocked in 1% BSA. After blocking, cells were stained of *ICP4* and *ICP8*. The nucleus was stained with DAPI and the green foci to indicate the presence of HSV-1protein.

To investigate the effect of *R. tanguticum* nanoparticles on HSV-1 genes expression, we examined the mRNA levels of HSV-1 immediate-early (IE), early (E) and late (L) genes by RT-qPCR. As shown in **Figure 5B**, the HSV-1 infected HEp-2 cells were incubated with or without *R. tanguticum* nanoparticles (350 µg/ml) at different time point (6, 12, 18, and 24 h) and the results showed that the mRNA levels of the IE gene *ICP4*, E gene *ICP8* and L gene *gD* were significantly reduced in a time-dependent manner when treated with *R. tanguticum* nanoparticles. *ICP4* is a transcription activator that is an essential factor for early and late promoter, while *ICP8*, the viral major DNA-binding protein and an E protein, is necessary for viral DNA replication. Therefore, the effect of *R. tanguticum* nanoparticles on their production was further determined by Western blot analysis and IFA. As shown in **Figure 5A**, the western blot results showed that *ICP4* and *ICP8* fails to express in virus-infected *R. tanguticum* nanoparticles treated cells which were significantly lower than in the virus-infected group. Besides, the results of IFA indicated that although the expression levels of *ICP4* treated with *R. tanguticum* nanoparticles at 350 µg/ml were similar to the virus control at 6 and 12 h.p.i., *ICP4* production was strongly suppressed by *R. tanguticum* nanoparticles at 18 and 24 h.p.i. (**Figure 6A**). At the same time, the production of *ICP8* in the virus control group was significantly more abundant than that in the *R. tanguticum* nanoparticles-treated cells at 18 and 24 h (**Figure 6B**). These results are consistent with those found and indicated that *R. tanguticum* nanoparticles could strongly affect HSV-1 replication through interfering with viral IE, E, and L gene expressions.

### *R. tanguticum* Nanoparticles Increases the Survival Rate of HSV-1 Infected Mice

**Figure 7:**
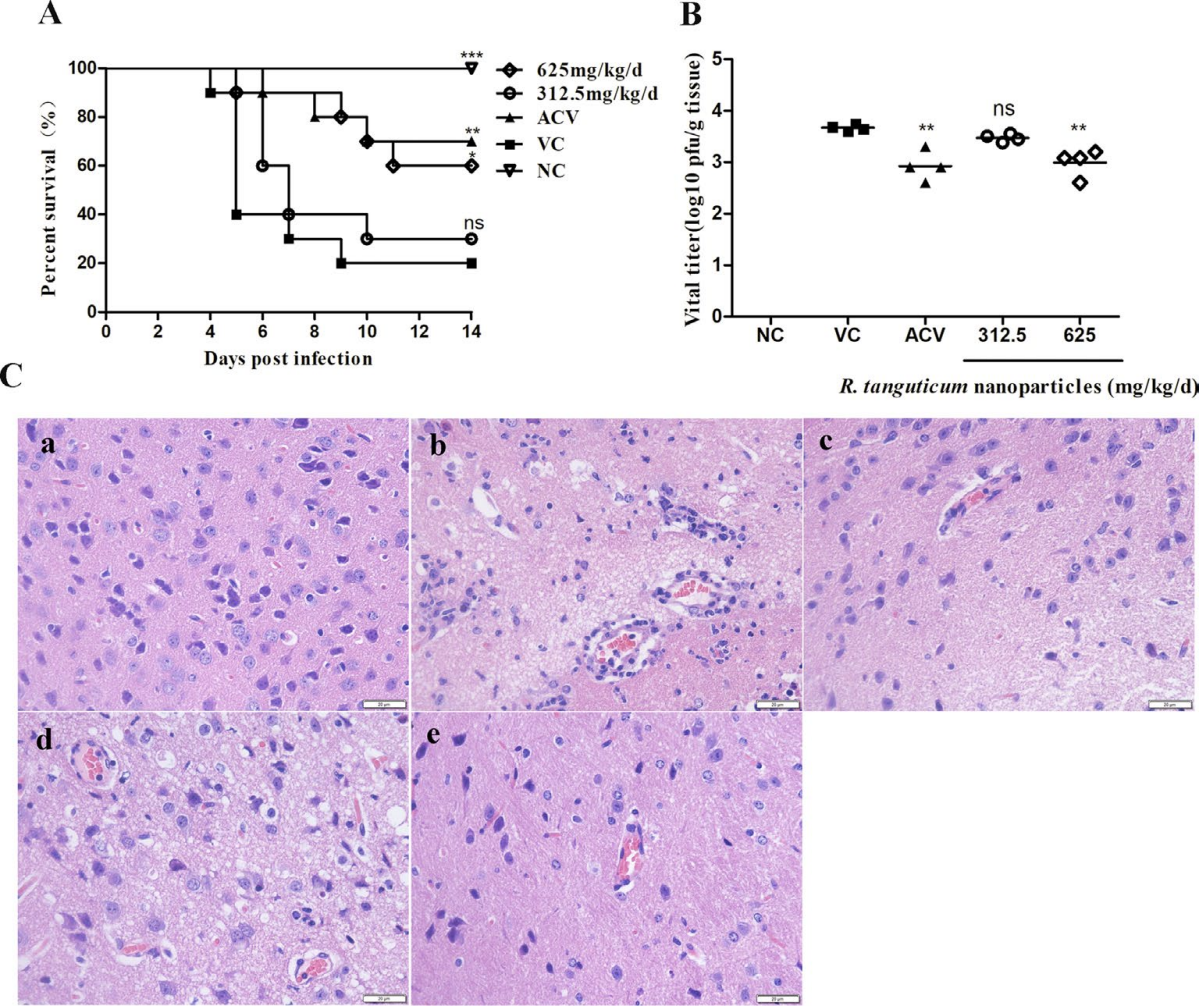
*R. tanguticum* nanoparticles alleviated HSV-1 infection in mice. Kunming mice (n = 10 mice/group) infected with 5 LD_50_ of HSV-1 were orally administered with 625 mg/kg/day and 312.5 mg/kg/day *R. tanguticum* nanoparticles, respectively. A 0.9% saline was used in viral control and normal control group. **(A)** The survival rates were collected daily for 14 day. **(B)** Brain Virus titers were determined by plaque assay at the 5th d.p.i. **(C)** Histological observations of cerebral tissues for mice sacrificed at the 5th d.p.i. Scale bar = 20 µm. (a) Mock infected mice treated with 0.9% saline (normal control, NC); (b) HSV-1-infected mice treated with 0.9% saline (viral control); (c) HSV-1-infected mice treated with acyclovir; (d–e) HSV-1-infected mice treated with 312.5 or 625 mg/kg/day of *R. tanguticum* nanoparticles.*p < 0.05; **p < 0.01; ***p < 0.001; n.s., not significant.

The experimental mice were monitored for 14 successive days. Clinical signs of changes in behavior of murine HSV-1 infection, such as lack of appetite hyperactivity and weight loss, a hunched posture, and inactivity were observed. The survival time, lethality and viral titers in the brains were recorded to determine the antiviral efficiency of *R. tanguticum* nanoparticles. As shown in **Figure 7A**, the survival rates of the *R. tanguticum* nanoparticles-treated groups at dosages of 312.5 mg/kg/day and 625 mg/kg/day were 30.0% and 60.0%, which are higher than the viral control group (20.0%) on the 14th day post-infection (d.p.i.). However, there was no significant difference of weight loss between all drug-treated groups and the virus control group (*p* > 0.05) (**Supplementary Figure S1**). Compared with the virus control group, *R. tanguticum* nanoparticles treated mice died later and the progress time course of clinical signs was slowed. Additionally, the protective rate of acyclovir at 100 mg/kg/day was shown to be 70%. The changes of the virus titers at the 5th d.p.i. were also detected to confirm the protective efficacy of *R. tanguticum* nanoparticles against HSV-1infection *in vivo*. Oral gavage by *R. tanguticum* nanoparticles in 625 mg/kg/day significantly reduced the virus titers compared to the virus control group (*p* < 0.01) (**Figure 7B**). Moreover, the treatment with *R. tanguticum* nanoparticles (625 mg/kg/day) showed no significant difference with the acyclovir group. Taken together, the results suggest that *R. tanguticum* nanoparticles could protect mice from HSV-1infection and prolong the lifespan.

### *R. tanguticum* Nanoparticles Decreased the Severity of Viral Brain Lesions Induced by HSV-1 Infection

We examined the pathological changes in mice brains harvested at the 5th d.p.i. In virus group, HE staining showed tissue hyperemia, inflammatory cell aggregation, degeneration of neural cells and virus-induced diffuse necrosis [**Figure 7C** (b)]. Following *R. tanguticum* nanoparticles (625 mg/kg/day) or acyclovir intervention, the damage of brain lesion was relieved and only mild tissue hyperemia could be found [**Figure 7C** (c and e)]. Furthermore, the level of neuronal degeneration and aggregation of inflammatory cell in brain tissue were significantly lower than that in virus group. However, the histological changes with the treatment with *R. tanguticum* nanoparticles at 312.5 mg/kg/day are similar to those in the virus control group [**Figure 7C** (d)]. These results indicated that post-treatment with *R. tanguticum* nanoparticles (625 mg/kg/day) can effectively alleviate the severity of the brain damage in the virus-infected mice.

### *R. tanguticum* Nanoparticles Can Suppress the Transcription Levels of *ICP4* and *ICP8* and Protein Levels of *ICP4* in the Brain Tissues of the Infected Mice

**Figure 8:**
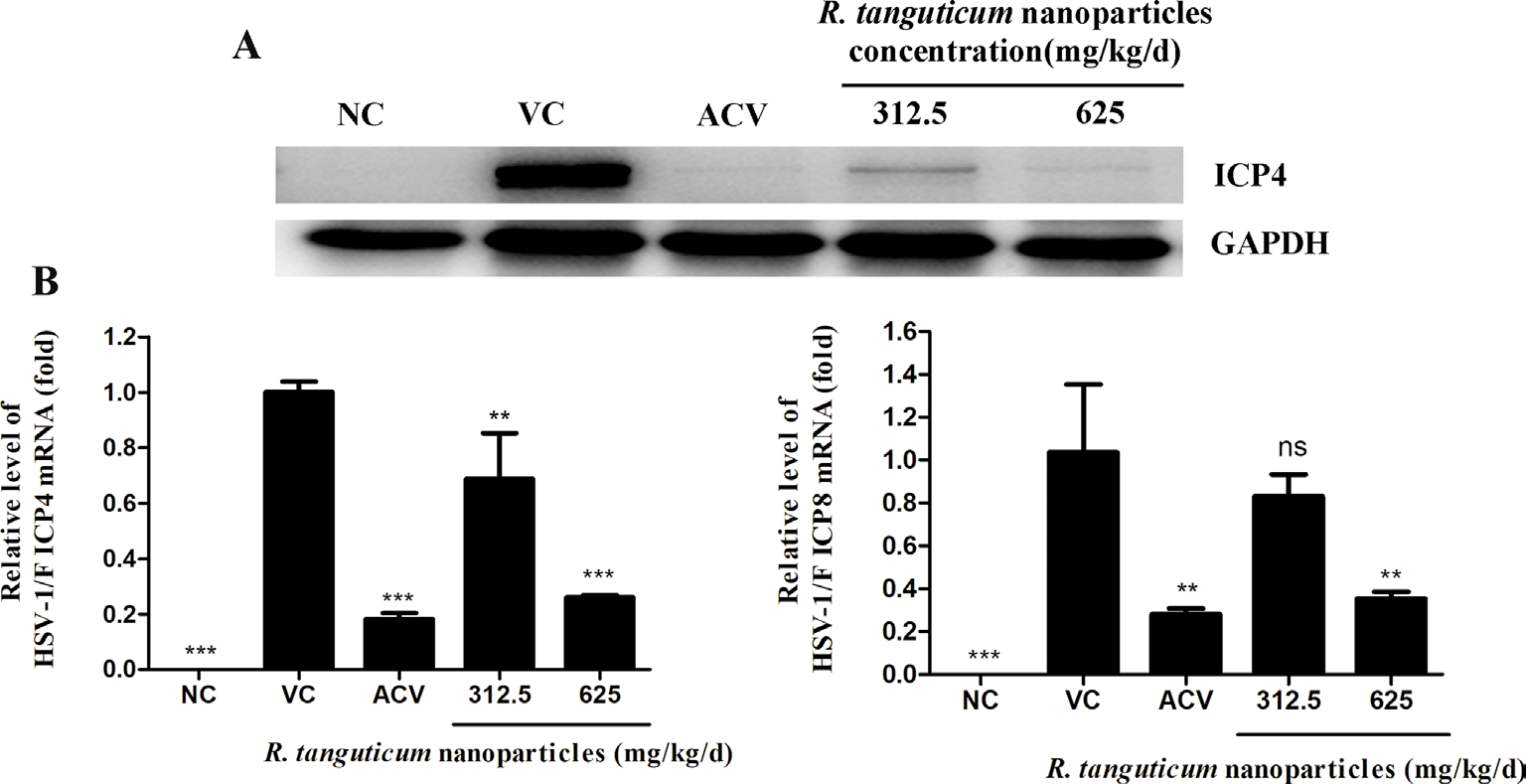
*R. tanguticum* nanoparticles treatment reduced the genes and protein expression in HSV-1 infected mice. Kunming mice (n = 4 mice/group) infected with 5 LD_50_ of HSV-1 were orally administered with 625 mg/kg/day, 312.5 mg/kg/day *R. tanguticum* nanoparticles, respectively. A 0.9% saline was used in viral control and normal control group. The mice were scheduled for sacrifice at 5 d.p.i. **(A)** The expression of the *ICP4* protein of the brain tissue was detected by Western blot analysis. **(B)** The mRNA levels of the *ICP4* and *ICP8* in treated and control groups were determined by real-time quantitative PCR analysis.*p < 0.05; **p < 0.01; ***p < 0.001; n.s., not significant.

We then assessed the expression of the *ICP4* and *ICP8* at mRNA levels *in vivo*. The result showed that when treated with *R. tanguticum* nanoparticles in 625 mg/kg/day, the mRNA expression levels of both *ICP4* and *ICP8* could be down-regulated in brain tissues of mice compared to virus control (*p* < 0.01) (**Figure 8B**). The groups treated with 312.5 mg/kg/day *R. tanguticum* nanoparticles could reduce the amount of *ICP4* mRNA levels (*p* < 0.01), while it showed no effect on the expression levels of *ICP8* with respect to the expression in virus control. The brain tissues were also analyzed to determine the expression levels of the *ICP4* protein by western blot. As shown in **Figure 8A**, treatment with *R. tanguticum* nanoparticles (625 mg/kg/day) significantly decreased the expression of the *ICP4* protein levels compared with non-treated infected groups. These results suggested that *R. tanguticum* nanoparticles could alter the transcription levels and suppress the expression of the virus protein *in vivo*.

## Discussion

Nanoparticles have been widely studied for their applications in fields such as drug delivery systems and antimicrobial agents (Singh and Nalwa, 2011). Moreover, nanoparticles exists broad-spectrum antiviral activity for its potential multi-targeting mechanism of action. It has been reported that silver nanoparticles have proven to be active against several types of viruses including human immunodeficiency virus, hepatitis B virus, herpes simplex virus, respiratory syncytial virus, and monkey pox virus (Galdiero et al., 2011).

Clinically, due to the large amount dosage use of traditional Chinese medicine with its poor effect of the ethanol or water extracts, ultrafine pulverizing technology, such as cryogenic grinding and air classifying grinding is applied to produce ultrafine powder (Li et al., 1991; Choi et al., 2012). It helps to increase the dissolution and improve the utilization ratio of the bioactive ingredients by cell breaking treatment (Chen et al., 2016; Xing et al., 2017). However, particle size distribution of their ultrafine powders only reaches to micron level. In our present study, the size of *Rheum palmatum* ultrafine powders was about to nanoscale. The preparation of *R. tanguticum* nanoparticles could increase the absorption of the effective ingredient and reduce dosage.

In this study, we demonstrated the antiviral activity of *R. tanguticum* nanoparticles against herpes simplex virus type I *in vitro* and *in vivo*. Virus inactivation assay showed that treatment of high concentration (350 µg/ml) of *R. tanguticum* nanoparticles can inhibit the formation of viral plaque by fifty percent approximately. This result indicates that *R. tanguticum* nanoparticles were likely to interact with cell-free virions, and this interaction results in suppression of viral infection. The inhibitory effect could also be observed from adsorption and penetration assays, which suggests that *R. tanguticum* nanoparticles could block the viral attachment and entry into cells. Further studies have found that *R. tanguticum* nanoparticles exerted a more effective antiviral activity when they were added post infection. The formation of viral plaques was reduced by fifty percent when treated with 194.1 µg/ml *R. tanguticum* nanoparticles, and plaques were completely inhibited when the concentration is 300 µg/ml or higher. The result of time of addition assay showed that more than 90% inhibition could be obtained even when the treatment was implemented 15 h post infection, suggesting that *R. tanguticum* nanoparticles could interfere with the entire phase of viral replication. The inhibitory activity on viral replication was further confirmed by evaluating the down-regulation of viral gene expression levels, including *ICP4* and *ICP8* that are essential factors for early and late promotor and viral gene expression. Taken together, our results indicate that *R. tanguticum* nanoparticles could inhibit HSV-1 infection by interfering with multiple steps of the viral life cycle, including attachment, entry and replication.

In addition, the efficacy of *R. tanguticum* nanoparticles was monitored *in vivo* using a mice encephalitis model caused by HSV-1 infection. The *R. tanguticum* nanoparticles (625 mg/kg/day) treated mice showed a better survival rate, decreased viral titers and alleviated clinical signs compared to the virus control group, and this efficacy was close to that of acyclovir (100 mg/kg/day). Our previous study showed that the ethanol extracts of *Rheum tanguticum*, emodin inhibit the herpes simplex virus *in vitro* and *in vivo*, which the most effective dose orally administered to mice was 6.7 g/kg/day (Xiong et al., 2011). Others have been reported that the rhubarb ethanol extract exhibited anti-viral effects on herpes simplex virus infection *in vivo* and they treated mice by injected hypodermically with the dose at 4.9 g/kg/day (Wang et al., 2003). In our present study, the effective dose of *R. tanguticum* nanoparticles that satisfies the efficacy may be reduced comparing to the previous studies. Moreover, compared with traditional ethanol or water extracts, *R. tanguticum* nanoparticles is a whole ingredient medicine maximized preserving the biologically active compositions without heating or damaging them. This indicates that nanoparticles could improve the pharmacokinetics and bioavailability of therapeutic agents.

It has been well documented in the literature that a variety of metal nanoparticles, especially the silver nanoparticles, exist efficient inhibitory activity against various viruses (Rai et al., 2016). Possible antiviral mechanisms of metal nanoparticles correspond to the stages of viral infection, including direct inactivation of cell-free virions (Cagno et al., 2018), interference with viral adsorption/penetration into the cell (Mehrbod et al., 2009; Hang et al., 2015), interaction with viral genome, inhibition of viral replication (Lu et al., 2008), inhibition of assembly and release of progeny virions.

In this study, the cell-free virion lost its infectivity to some extent when incubated with *R. tanguticum* nanoparticles pre-infection, indicating that *R. tanguticum* nanoparticles could interact with virion. However, whether the interaction could destroy the virion structure or just block the receptor binding site on the viral envelope is not clear. Further experiments need to be carried out to confirm which way the *R. tanguticum* nanoparticles work by using transmission electron microscopy (Cagno et al., 2018). Heparin sulfate proteoglycans (HSPGs) on the cell surface is involved in the infection of many viruses, including herpes viruses, for it can serve as primary receptors or co-receptors for pathogens (Liu and Thorp, 2002). HSV glycoproteins gB and gC bind to cell-surface HSPGs and mediate the initial attachment to target host cells (Laquerre et al., 1998). Lara et al (Lara et al., 2010) reported that silver nanoparticles were capable of interfering with gp120-CD4 interaction and result in the blockage of viral entry into cells. This result inspires us that *R. tanguticum* nanoparticles might interfere with the gB/gC-HSPGs interaction. A competitive gB/gC-capture ELISA using HSPGs-coated plate should be performed to verify this hypothesis. Dey et al (Dey et al., 2018) stated that they visualized the uptake of nanogels by the cells through clathrin-mediated endocytosis using confocal microscopy. It indicated that nanoparticles might get access into the cell and inhibit viral replication. Thus, the inhibitory effect of *R. tanguticum* nanoparticles on viral gene transcription and translation of viral proteins might be due to interactions of *R. tanguticum* nanoparticles with viral genome, certain factors or pathways essential for viral replication. However, the precise mechanism needs to be further investigated.

Previous studies have shown that the size of nanoparticles could affect the antiviral activity. Rogers et al. (Rogers et al., 2008) reported that silver nanoparticles with diameters between 10 to 80 nm exhibited an antiviral activity against the monkeypox virus. Their results indicate that the silver-containing nanoparticles with a diameter of approximately 10 nm (Ag-PS-10) were the most effective at inhibiting MPV infectivity. In addition, study by Orlowski et al (Orlowski et al., 2014) demonstrated that the smaller-sized nanoparticles could induce production of cytokines and chemokines that important for anti-viral response. Nonetheless, one should keep in mind that various *in vitro* and *in vivo* studies demonstrated the nanosilver-related toxic effects in rat hepatocytes and neuronal cells (El Mahdy et al., 2015), human lung epithelial cells (Pinzaru et al., 2018) and rat’s living tissues (Sardari et al., 2012). In our present study, TEM image of *R. tanguticum* nanoparticles showing the particle size range from 50–219 nm. Nanoparticles with large sizes are likely to interact with virions and inactivate the virions or block the viral attachment and entry into cells, whereas smaller nanoparticles might get access into the cell and interact with viral genome, certain factors or pathways essential for viral replication.

Plants-derived silver nanoparticles have been extensively studied for their antiviral efficacy. Salem et al. (Ben Salem et al., 2012) reported for the first time that the silver nanoparticles synthesized from *Ricinus communis* leaf and fruit extracts showed great antiviral activity against Coxsackievirus B3 when both incubated with cell culture and virus suspension. Another study stated that the silver nanoparticles derived from *Cinnamomum cassia* extracts exhibited an enhancement of antiviral activity against highly pathogenic avian influenza virus subtype H7N3 as compared with *Cinnamomum cassia* extracts itself (Fatima et al., 2016). In addition, green synthesis of silver nanoparticles using *Rheum palmatum* root extracts has been reported by Arokiyaraj et al (Arokiyaraj et al., 2017) recently. The particles were spherical and hexagonal shapes with an average size of 121.5 nm, and showed an excellent bactericidal effect against *S. aureus* and *P. aeruginosa*. However, the antiviral activity of these nanoparticles was not evaluated at that time. Noting that their biosynthesis particle’s sizes are similar to these physically synthesized nanoparticles used in our present study, we believe that the antiviral activity of our nanoparticles is authentic.

Above all, the findings reported here provide novel insights into the antiviral activity of *R. tanguticum* nanoparticles and offer a new and promising way for the usage of Chinese herbal medicine to control the infection caused by pathogenic microorganisms.

## Data Availability

The datasets generated for this study are available on request to the corresponding author.

## Ethics Statement

This study was carried out in accordance with the recommendations of the Chinese Animal Protection Act and the National Research Council criteria. The protocol was approved by the Ethics Committee of Wuhan University School of Medicine.

## Author Contributions

M-xS, H-rX, and Z-qY conceived and designed the experiments. M-xS, NM, M-kL, Y-yL, and TC performed the research. FW, D-yL, M-xS and WH analyzed the data. M-xS, H-rX, and Z-qY wrote the paper.

## Funding

This work was supported by the Major Program for Technique Development Research of Novel Medicine Production (No. 2009ZX09301) and the National Natural Science Foundation of China (NSFC project No. 30873104).

## Conflict of Interest Statement

The authors declare that the research was conducted in the absence of any commercial or financial relationships that could be construed as a potential conflict of interest.
