# Supplementary material for: Antiviral Properties of R. tanguticum Nanoparticles on Herpes Simplex Virus Type I In Vitro and In Vivo
Source: Front Pharmacol. 2019 Sep 4;10:959. doi: 10.3389/fphar.2019.00959 (PMC6737004; doi:10.3389/fphar.2019.00959)
Supplement: Supplementary file 1 [file Table_1.docx]

**Supplementary figures**

**
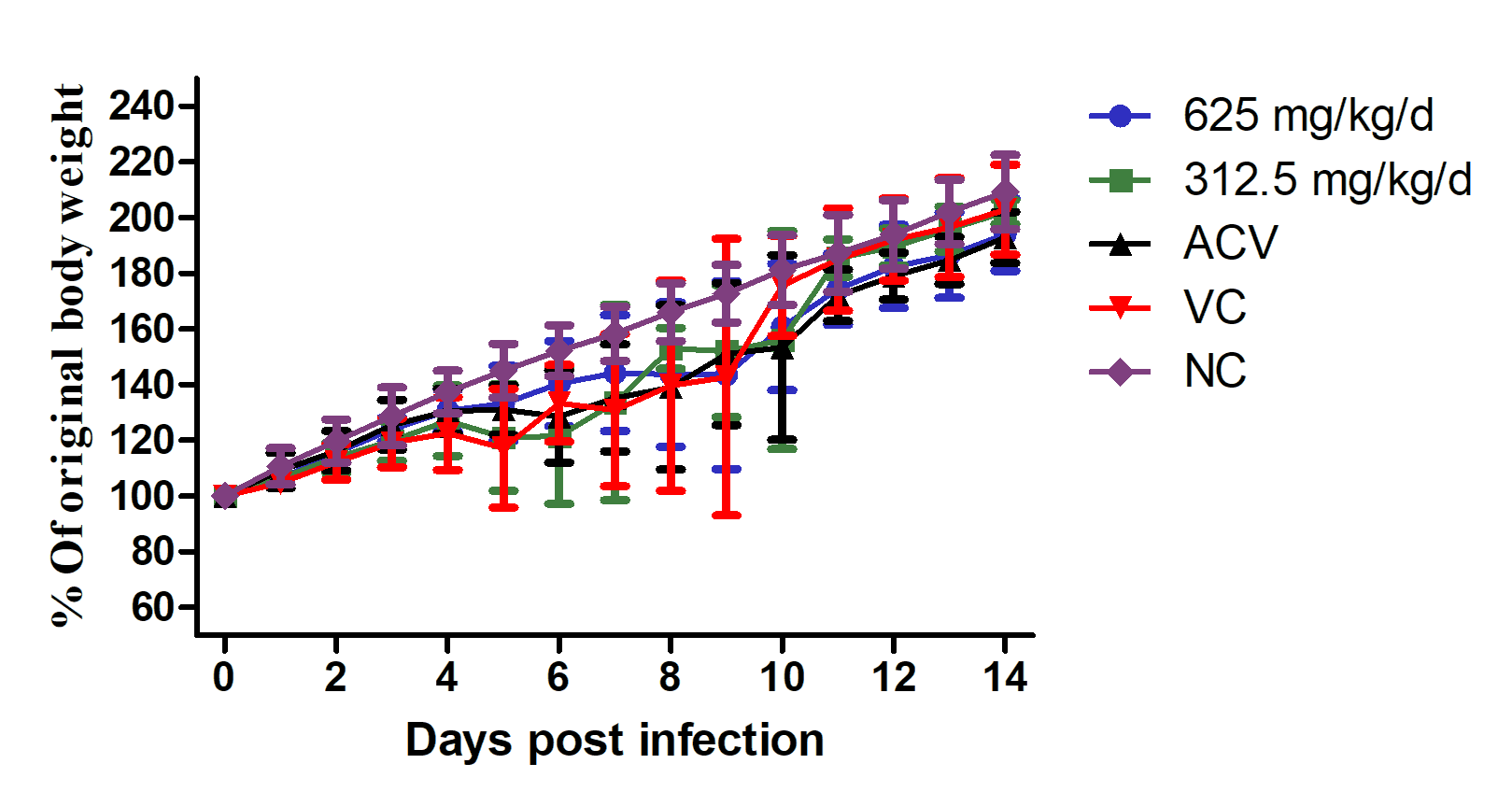
**

**Figure S1**. ***R. tanguticum* nanoparticles alleviated HSV-1 infection in mice.** Kunming mice (n = 10 mice/group) infected with 5 LD_50_ of HSV-1 were orally administered with 625 mg/kg/d, 312.5 mg/kg/d *R. tanguticum* nanoparticles, respectively. 0.9% saline was used in viral control and normal control group. The body weight of each groups were determined.*, *p* < 0.05; **, *p* < 0.01; ***, *p* < 0.001; n.s., not significant.
